# Sequence Transpositions Restore Genes on the Highly Degenerated W Chromosomes of Songbirds

**DOI:** 10.3390/genes11111267

**Published:** 2020-10-28

**Authors:** Luohao Xu, Martin Irestedt, Qi Zhou

**Affiliations:** 1 Department of Neurosciences and Developmental Biology, University of Vienna, 1090 Vienna, Austria; zhouqi1982@zju.edu.cn; 2 Department of Bioinformatics and Genetics, Swedish Museum of Natural History, 104 05 Stockholm, Sweden; martin.irestedt@nrm.se; 3 MOE Laboratory of Biosystems Homeostasis & Protection, Life Sciences Institute, Zhejiang University, Hangzhou 310012, China; 4 Center for Reproductive Medicine, The 2nd Affiliated Hospital, School of Medicine, Zhejiang University, Hangzhou 310012, China

**Keywords:** avian genomics, sex chromosomes, transpositions, sex-specific selection

## Abstract

The female-specific W chromosomes of most Neognathae birds are highly degenerated and gene-poor. Previous studies have demonstrated that the gene repertoires of the Neognathae bird W chromosomes, despite being in small numbers, are conserved across bird species, likely due to purifying selection maintaining the regulatory and dosage-sensitive genes. Here we report the discovery of DNA-based sequence duplications from the Z to the W chromosome in birds-of-paradise (Paradisaeidae, Passeriformes), through sequence transposition. The original transposition involved nine genes, but only two of them (*ANXA1* and *ALDH1A1*) survived on the W chromosomes. Both *ANXA1* and *ALDH1A1* are predicted to be dosage-sensitive, and the expression of *ANXA1* is restricted to ovaries in all the investigated birds. These analyses suggest the newly transposed gene onto the W chromosomes can be favored for their role in restoring dosage imbalance or through female-specific selection. After examining seven additional songbird genomes, we further identified five other transposed genes on the W chromosomes of Darwin’s finches and one in the great tit, expanding the observation of the Z-to-W transpositions to a larger range of bird species, but not all transposed genes exhibit dosage-sensitivity or ovary-biased expression We demonstrate a new mechanism by which the highly degenerated W chromosomes of songbirds can acquire genes from the homologous Z chromosomes, but further functional investigations are needed to validate the evolutionary forces underlying the transpositions.

## 1. Introduction

Unlike mammals, birds have a female heterogametic (ZW) sex chromosome system, i.e., females have a female-specific W chromosome and a Z chromosome, whereas males have two Z chromosomes. Similar to the mammalian Y chromosomes, the avian W chromosomes are highly degenerated and gene-poor, except for palaeognathous birds [1,2], suggesting convergent sex chromosome degeneration [3]. The degeneration of the W chromosomes makes the Z chromosome hemizygous throughout most of its length in females, with only a handful of genes surviving on the W chromosomes (those homologous ZW gene-pairs are called gametologs) [4,5]. For instance, a typical Z chromosome of Neognathae birds has more than 700 genes, but the W chromosome has only ~40 intact genes; in other words, more than 90% of the original gene content has been eliminated from the W chromosomes [5,6].

Characterization of the survivor genes on the Y and W chromosomes, therefore, is useful to elucidate the evolutionary forces that govern the retention of genes on the decaying sex chromosomes. Previous studies on chicken [7] and songbird [6] W chromosomes suggest a common force that shapes the gene content of the W chromosome, that is, purifying selection retaining the dosage-sensitive or housekeeping genes. This is consistent with what has been found for the mammalian Y chromosomes [8] and snake W chromosomes [9], though other genetic constraints on the mammalian Y chromosome have been recently proposed [10]. For the avian W chromosome, however, the strength of purifying selection on dosage-sensitive genes is perhaps stronger because the global dosage compensation is lacking or incomplete in birds [11,12].

In many organisms, however, the gene content of the Y chromosomes is more than simply the outcome of degeneration from the proto-Y chromosome. For instance, most of the *Drosophila* Y-linked genes were duplicated from the autosomes and are testis-specific [13,14], through DNA-based transposition [15] or RNA-based retrotransposition [16], likely driven by male-specific selection. Recruitment of genes into the Y chromosome through retrotransposons [17,18,19] and X-linked transposition [20,21,22,23,24] have also been widely reported across mammalian species. The Y-borne genes often massively increase their gene copies through amplification, which has been documented in both mice [25] and *Drosophila* [26]. The amplification can occur independently in multiple mammalian and Drosophila lineages, resulting in notable differences in gene content of the Y chromosomes even between closely related species [27,28,29]. Additionally, the genetic information of the Y chromosomes can sometimes be influenced by X-Y gene conversion by which the coding sequence of the Y-linked genes can be rewritten using that of the X-linked copy, retarding the genetic decay of the Y-linked genes. This evolutionary mechanism has been observed across a broad range of mammalian species [30,31,32]. Finally, gene conversion can also happen between the inverted repeats (palindromic sequences) of the Y chromosome, and such intra-chromosome gene conversion has been frequently found to maintain the intactness of coding genes within the palindromes [33,34]. Those observations suggest the action of multiple evolutionary forces that impact the gene content of the Y chromosome, with the male-specific selection as the dominant one.

On the contrary, previous studies suggested that the avian W chromosomes have relatively more conserved and evolutionarily stable gene content across Neognathae [6,7], covering the majority of bird species. This is partly because Neognathae shares a large portion (~50%) of non-recombining regions along the sex chromosomes and the majority of original genes had been deleted prior to species radiations [6]. Despite the overall conservation, the alteration of gene content on the avian W chromosomes has also been observed in a similar way as in *Drosophila* and mammals, with some differences. For example, *HINT1* gene has been amplified on the chicken W chromosomes [7], though the copy number is limited to two or three in songbirds [6], and its expression does not seem to be ovary-biased [5,7]. In a rare case, an autosome-derived retrotransposed gene *NARF* has been found on the W chromosome of a crow [6], but it is not a gene with female-biased expression. To date, while intra-chromosome gene conversion seems to have occurred in New World sparrows and blackbirds [35], no transposition or Z-W gene conversion has been documented in bird sex chromosomes. It has been demonstrated that female-specific selection can drive the expression upregulation of W-linked genes in chicken breeds [36], but it is unclear whether it can recruit new genes into the W chromosomes. In this study, we report transpositions from the Z to W chromosomes across three lineages of songbirds that resulted in the restoration of several once-lost gametologs onto the W chromosomes. We found the transposition of at least one gene (*ANXA1*) was likely favored by female-specific selection, and more than half of the transposed genes were predicted to be dosage-sensitive; therefore, their restoration onto the W chromosome may help resolve the gene dosage imbalance.

## 2. Materials and Methods

### 2.1. Identification of Z-Linked Transpositions

The genomic and resequencing data used in this study are listed in Supplementary Tables S1 and S2. For the 12 songbird genomes, genomic data are available for both sexes except for three species. We first used the published Z chromosome sequence of great tit [37] to identify and order the Z-linked sequences among the investigated species. To calculate the sequencing coverage, we mapped the reads to the reference genomes using BWA-MEM (0.7.16a-r1181) with default parameters. We used the “depth” function in samtools (1.9) [38] to calculate coverage for every nucleotide site, subsequently removed those sites with mapping quality (-Q) lower than 60 or depth 3 times higher than average. Then we calculated genomic coverage of every 50 kb sliding window by using the “bedtools map” function. Any windows with less than 60% of the region (30 kb) mapped by reads were excluded. We used the GATK (3.8.0) [39] pipeline (HaplotypeCaller) to call variants. Raw variants were filtered by these criteria:—window 10—cluster 2 “FS > 10.0”, “QD < 2.0”, “MQ < 50.0”, “SOR > 1.5”, “MQRankSum < −1.5”, “ReadPosamplenkSum < −8.0”. We further required the variants showing an allele frequency between 0.3 and 07 (the expected heterozygosity should be 0.5 for one individual, but we allowed for some variation). The SNP density was defined by the number of SNPs over a 50 kb window.

Because the transpositions were inferred to be evolutionarily young (based on the inferred ages) and the W-derived reads can perfectly map to the Z-linked sequences, we used the called variants (both SNP and indels) from female reads to create the pseudo-sequence of the W-linked ZTR. To do so, we applied the FastaAlternateReferenceMaker tool from the GATK package. The gene models on the W chromosomes were then predicted by genewise (2.4.1) [40] with the protein sequences of the Z-linked homologs. To remove potential chimeric W-derived alleles in the Z-linked regions (due to the collapse of genome assembly), if any, we used male sequencing reads to polish the Z-linked sequence using pilon (1.22) [41].

To verify the identified ZTR in the great tit, we randomly chose 10 sequence fragments containing multiple SNP sites for designing primers and performed PCR amplification. The information about PCR primers were listed in Supplementary Table S3. The PCR products were purified and sequenced at Macrogen (Macrogen, Amsterdam, The Netherlands, Europe B.V.).

### 2.2. Synonymous and Nonsynonymous Substitution Rate

We aligned the Z- and W-linked ZTR genes of interests and several outgroups species (Supplementary Table S1) using the GUIDANCE2 (2.02) [42] pipeline. PRANK (170427) [43] was used as the aligner. Then we used the codeml tool from the PAML package (4.9e) [44] to estimate the substitution rate. We used the codeml model that allowed the substitution rate to vary among the branches.

### 2.3. Haploinsufficiency Score

We measured the probability of haploinsufficiency of avian genes, based on the published haploinsufficiency scores [45] of their human orthologs. Haploinsufficiency score is defined as the sufficiency of one single copy of genes to accomplish normal gene functions. Huang et al. predicted haploinsufficiency score for each human gene, based on known haploinsufficient genes identified from disease studies, and haploinsufficient genes having copy number variations among healthy human individuals.

### 2.4. Gene Expression Analyses

We used the program RSEM [46] (1.3.0) pipeline to estimate gene expression levels. We downloaded the RNA-seq data from the great tit [37], blue tit [47], collared flycatcher [48], rock pigeon [49,50], guineafowl [51], goose [51], duck [51], turkey [51], chicken [52], Chilean tinamou [1] and green anole [53], across tissues of both sexes (gonad, brain, liver, spleen and heart) when available. The accession numbers are listed in the Supplementary Table S4. When biological replicates were present, we downloaded RNA-seq data of all replicates, and the expression levels were calculated as the mean over replicates. The details of the methods of RNA-seq read mapping and expression level quantification were described in [6].

### 2.5. Data Accessibility

The published genomes and sequencing reads are listed in Supplementary Tables S1, S2 and S4. Codes have been deposited at GitHub [54].

## 3. Results

### 3.1. Discovery and Characterization of Sequence Transposition in Birds-of-Paradise

**Figure 1:**
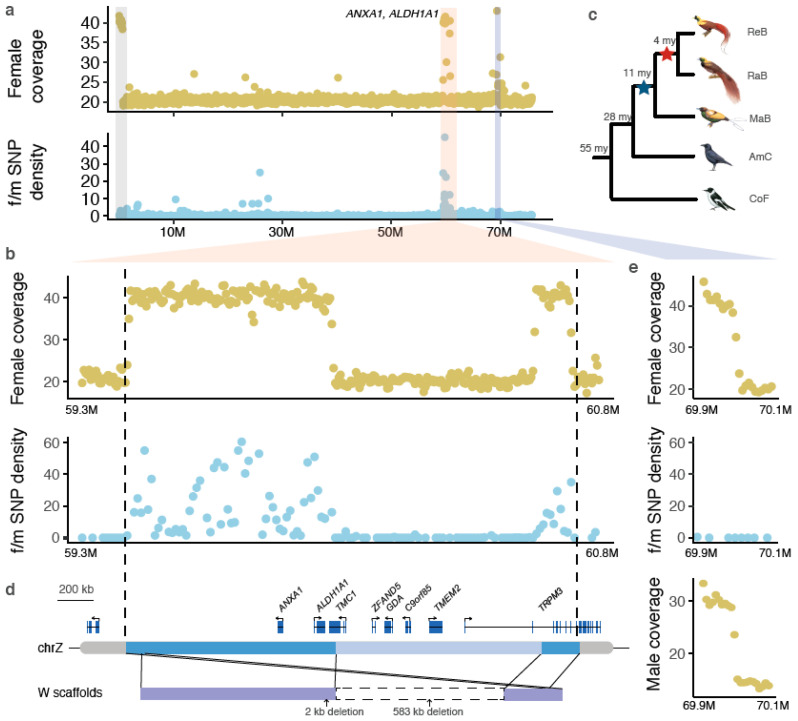
A transposition from the Z to W chromosomes in birds-of-paradise. (**a**) the genomic region at ~60 Mb of the Z chromosome showing doubled female sequencing coverage and female-specific elevations of heterozygosities (“f/m SNP density”) was inferred as a recent Z-transposed region (ZTR), and is marked by a red vertical bar. Two genes that survived following ZTR were labeled close to the red vertical bar. PAR and Z-linked duplications (marked in gray and purple vertical bars) are not expected to show a female-specific elevation of SNP densities. (**b**) A zoom-in view of the ZTR, showing female coverage and female-to-male SNP density calculated in 50 kb windows. (**c**) The phylogeny of birds-of-paradise (BOP) showing the origin of the ZTR (red asterisk) and the Z-linked duplication (blue asterisk). ReB: red BOP, RaB: Raggiana BOP, Mab: magnificent BOP, AmC: American crow, CoF: collared flycatcher. All bird illustrations were ordered from https://www.hbw.com/. (**d**) The 1.3 Mb transposed sequence (vertically aligned with (B)) contains 8 genes, but 4 complete and 2 partial genes have been deleted due to a 583 kb long deletion. Only *ANXA1* and *ALDH1A1* were retained on the W after transposition. (**e**) A zoom-in view for the duplication that does not show elevated female-to-male SNP density and both female and male coverage are doubled.

Because of the nearly complete degeneration of the W chromosomes in Neognathae birds (including songbirds), the Z chromosomes (except for the pseudoautosomal region, or PAR) are expected to be hemizygous and to have a reduced sequencing coverage in females. However, we found that one region residing at ~60 Mb of the Z chromosome of red bird-of-paradise (BOP, family Paradisaeidae, order Passeriformes) showed a female coverage level similar to that of autosomes or the PAR (Figure 1a,b). In this region, female reads also show a remarkably higher level of heterozygosity than male reads, indicating recent Z-W sequence divergence (Figure 1a,b, Supplementary Figure S1). This excludes the possibility of a Z-to-autosome translocation that might explain the autosome-like coverage pattern, because sex difference in heterozygosity is not expected for autosomes where the homologous chromosomes can freely recombine. We further excluded the possibility of Z-linked duplication, because we only found the doubled coverage in females but not in males (Supplementary Figure S2). Finally, the genomic scaffold that harbors this entire region shows highly conserved gene syntenies with the Z chromosome sequences of other songbirds, suggesting sequence assembly of this region is unlikely to be erroneous (Supplementary Figure S3). The remarkably high heterozygosity in females, equal coverage between males and females and the conserved synteny together suggest there is likely a new copy of this region recently transposed from the Z to the W chromosome followed by rapid Z-W divergence. Those patterns were observed in two *Paradisaea* BOP, but not in other BOP genera (Supplementary Figure S4), suggesting the origin of the Z-to-W transposition was prior to the divergence of *Paradisaea* BOP 4 million years ago (mya) (Figure 1c). Because the duplicated region involved a large genomic sequence spanning several complete genes (Figure 1d), the duplicative sequence movement should represent a result of DNA-based transposition rather than RNA-based retrotransposition. We named the W-linked region that is derived from the Z chromosome as the Z-transposed region (ZTR), following the naming of previously reported XTR (X-transposed region) in mammals.

It is noteworthy that our analysis also uncovered a recent duplication on the Z chromosome at ~70 Mb position (Figure 1a). It is different from ZTR in that male reads also showed increased coverage (Figure 1e). This inferred duplicated region (about 100 kb long) spans one gene *YTHDC2* and exhibits similar levels of read coverage and heterozygosity between males and females (Figure 1e). Similar to the case of transpositions, only one copy was assembled with short-reads because of the low sequence divergence between the two duplicated copies. Therefore, we rely on the read coverage to infer the increase of copy number. A recent long-read genome assembly of a basal BOP (*Lycocorax pyrrhopterus*) [55] has successfully assembled the two copies of this sequence (data not shown), suggesting the existence of this duplication and dating the duplication event further back to all BOPs (Figure 1c).

The newly duplicated sequence on the W chromosome is subject to decay upon its arrival on the W because of the absence of recombination. The length of the W-linked ZTR is 0.7 Mb, shorter than the parental Z-linked sequence (1.3 Mb), primarily due to a large deletion (583 kb) (Figure 1d). This large deletion removed five complete (*ZFAND5*, *TRPM3*, *TMEM2*, *GDA*, *C9orf85*) and 2 partial (*TMC1* and *TRPM3*) genes on the W-linked ZTR, accounting for 78% (7 out of 9) of the originally transposed genes (Figure 1d). Apart from that, we detected only two additional small sequence deletions (Supplementary Figure S5). The rapid sequence loss on the W chromosome is consistent with the general mode of sex chromosome degeneration at the early stage [6]. However, we did not detect any TE insertions on the W-linked ZTR.

**Figure 2:**
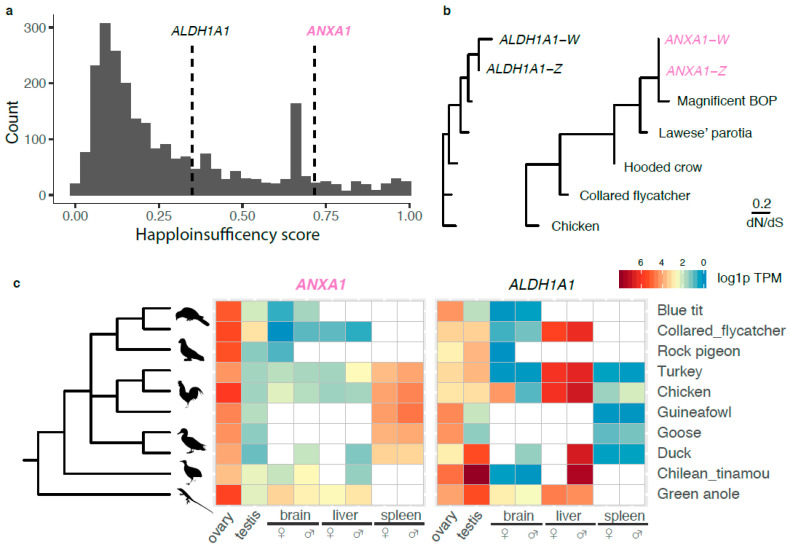
Transposed genes in birds-of-paradise are likely under evolutionary constraint (**a**) The distribution of predicted haploinsufficiency scores of human orthologs of all Z-linked genes, marked with ZTR genes *ALDH1A1* and *AXA1* by vertical dashed lines. (**b**) The phylogenetic tree with the branch lengths scaled by the ratios of nonsynonymous substitution rate to synonymous substitution rate (dN/dS). No nonsynonymous substitutions have been detected for *ANXA1*. (**c**) The expression of *ALDH1A1* and *ANXA1* across four different tissues of both male and female in nine birds and one reptile species. The expression levels were log1p (log 1 + TPM) transformed. Species that do not have the tissue transcriptome are denoted with blank tiles.

The retained W-linked ZTR contains only two intact genes: *ANXA1* and *ALDH1A1* (Figure 1d). Interestingly, both genes have high predicted haploinsufficiency scores (extrapolated from their human orthologs [45]), i.e., high dosage sensitivity (Figure 2a). While *ALDH1A1* shows a relatively relaxed evolutionary constraint measured by the ratio of nonsynonymous over synonymous substitution rates, *ANXA1* seems to be under strong purifying selection without a single nonsynonymous mutation in the W-linked copy (Figure 2b, Supplementary Table S5). In the absence of transcriptome data of BOPs, we used the RNA-seq data from other birds to infer the expression profiles of *ANXA1* and *ALDH1A1* prior to the transposition event in BOP. We found *ALDH1A1* has a broad and non-sex-biased expression pattern, similar to other W-linked gametologs [6,7], but *ANXA1* has a highly biased expression in ovaries, in all birds investigated here and a reptile (green anole lizard) (Figure 2c). Additionally, the ovary-biased expression of *ANXA1* is only seen in later developmental stages of females (Supplementary Figure S6). Together, these results suggest the new copy of *ANXA1* transposed onto the W chromosome is under strong purifying selection in females, potentially associated with its functional role in mature ovaries.

### 3.2. Identification of Sequence Transposition in Additional Songbird Lineages

**Figure 3:**
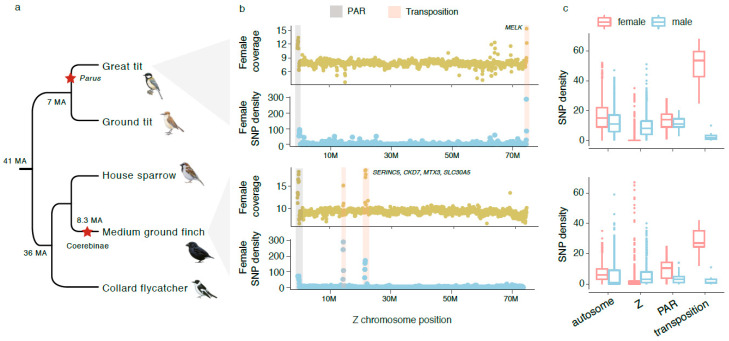
ZTRs identified in another two songbird lineages. (**a**) We detected ZTRs in great tit (*Parus*) and medium ground finch (Coerebinae). The red asterisks denote the origins of ZTRs. (**b**) The ZTR regions are highlighted by the vertical red bars, showing double female coverage and increased female SNP density. The grey bars highlight the PARs which have double female coverage but do not have increased female SNP density. The intact genes in the ZTRs are labelled near the red bars. (**c**) The ZTRs (transpositions) have significantly higher (Wilconxon rank sum test, *p* < 3.8 × 10^5^) female SNP density than the rest of the Z chromosome and autosomes, or than the male SNP density, for both species.

Having discovered the ZTR in BOPs, we set out to screen for signals of ZTR in other songbirds where both male and female sequencing reads are available and the Z-linked sequences have been previously identified [6]. Out of the five songbird species that we examined, we detected ZTRs in two species: medium ground finch and great tit (Figure 3). There are two ZTRs in medium ground finch, spanning about 100 and 200 kb of Z-linked homologous regions respectively, but only the second ZTR contains protein-coding genes (*SERINC5*, *CKD7*, *MTX3*, *SLC30A5*, *THBS4*) (Figure 3b). None of these five genes have been deleted on the W chromosome, but *THBS4* has probably become a pseudogene due to frameshift mutations (Supplementary Figure S7). By examining other Darwin’s finches and their relatives, we inferred that the ZTRs occurred and were fixed at the ancestor of Coerebinae (Darwin’s finches and their closest relatives) 8.3 mya, because the ZTRs are present in all Coerebinae species but not in Sporophilinae (Supplementary Figure S8). In great tit, the ZTR is located at the end of the Z chromosome, about 50 kb long, and contains one single gene *MELK* (Figure 3b). This ZTR is also present in another *Parus* species (Supplementary Figure S9), so it is likely that it has been fixed at the common ancestor of *Parus*. To further assess whether the ZTR is fixed in the great tit population, we randomly genotyped 10 fragments with 7 in the ZTR and 3 in the nearby regions for 6 female and 6 male great tit individuals, and found in the ZTR the Z-W divergence sites were all heterozygous in females but homozygous in male (Supplementary Figure S10), consistent with the pattern of Z-W divergence between the ZTR and its parental Z-linked copy.

**Figure 4:**
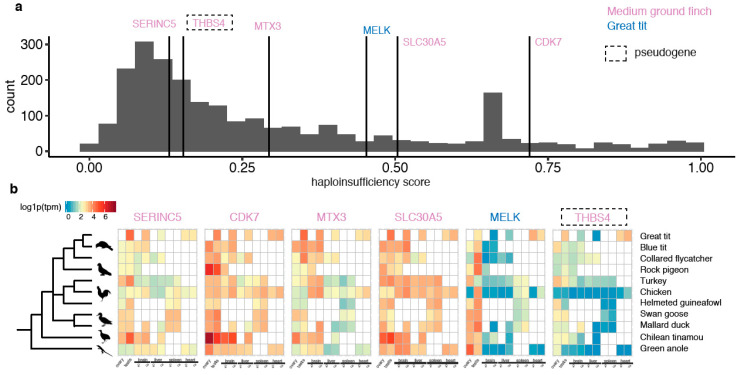
The predicted dosage-sensitivity and expression profile of the transposed genes. (**a**) The distribution of predicted haploinsufficiency scores of human orthologs of all Z-linked genes, marked with ZTR genes. *MELK*, *SLC30A5* and *CDK7* have relatively higher dosage-sensitivity (measured by haploinsufficiency scores) than the rest Z-linked genes. (**b**) The expression of the ZTR genes across tissues of both male and female in birds and a reptile. The expression levels were log1p (log 1 + TPM) transformed. The pseudogene was denoted by the dashed square.

Together with the two survivor genes in BOPs, we discovered in total seven novel W-linked genes in songbirds due to Z-to-W transpositions. Like *ANXA1* and *ALDH1A1*, *CDK7* and *SLC30A5* of medium ground finch and *MELK* of great tit are predicted to be dosage-sensitive relative to other Z-linked genes (Figure 4a). *CDK7* and *SLC30A5* are also broadly expressed at a relatively high level, but the expression of *MELK* seems to be biased in the gonads. The other two functional genes *SERINC5* and *MTX3* do not seem to be dosage-sensitive but are broadly expressed.

## 4. Discussion

In this study, we present the discovery and characterization of DNA-based sequence transpositions from the Z to the W chromosome in songbirds. In some other female heterogametic organisms including Lepidoptera [56] and plants [57,58], gene movement into the W chromosomes have been reported in recent years but not in birds. The bird genomes and chromosomes are characteristic for their evolutionary stasis with infrequent chromosomal rearrangements and gene movement [4,59]; consistently, the sex chromosomes and the gene content on the W chromosome remain stable over tens of million years’ evolution in birds [60]. The discovery of transpositions from the Z to W chromosome uncovers a new source from which the bird W chromosomes may regain once-lost genes.

Because population genetic data of BOPs is currently not available, we were only able to verify the transpositions using a molecular approach in the great tit. However, given the similar coverage and SNP density patterns exhibited by the ZTRs between the great tit and BOPs, it is reasonable to suggest the detected ZTR in BOPs is likely not an artifact. It needs to be noted that we have not directly assembled the sequences of W-linked ZTR genes, but because the transposition events are evolutionarily young (according to the ages we inferred) which allows the W-derived reads to map to the Z-linked sequences, we were able to infer the increase of copy number and the present of a new copy on the W chromosome. Our approach is not compatible with detecting ancient transposition events, if any. However, when the sequence of the W chromosome becomes available, the existence of ancient transposition can be directly tested. On the other hand, if ancient transposition events indeed existed, it raises the possibility that some of the gametologs might be the transposed (secondarily acquired) instead of the retained proto-W genes, particularly for those that are species- or lineage-specific. This can be detected through phylogenetic analyses on the Z and W gametologs [61,62], but it is beyond the scope of this study.

Undoubtedly, occasional transpositions are not expected to alter the evolutionary fate of the W chromosomes in Neognathae birds that are already gene-poor and highly degenerated, accompanied by massive accumulation of repeats [55,63]. The degenerative divergence of the W chromosome has made it distinct from the Z chromosome at both cytogenetic and sequence levels [4,6,64]. Additionally, when Z-to-W sequence transpositions occur, at a local scale, the newly arrived sequence onto the W chromosome is immediately subject to decay due to the lack of recombination. However, the duplicative transpositions give rise to intact genes on the W chromosomes, a result similar to gene conversion between the homologous sex chromosomes [30,31,32]. Importantly, when the transposed genes are beneficial to female individuals and when the female-specific selection is sufficiently strong, the transposed genes may survive in the “harsh” environment of the W chromosomes. Alternatively, the fixation of the transposed genes is simply because of genetic drift. Further investigations, including direct assembly of the W-linked ZTRs using long-read sequencing technology, and transcriptome and epigenetic profiling for the transposed genes in female organs, will provide more insights into the functional relevance of the ZTRs.

## 5. Conclusions

Among the 12 songbird genomes we investigated, five (in three lineages) exhibit signatures of sequence transposition from the Z to W chromosomes. The duplicative transposition is DNA based, and in all three bird lineages the transpositions led to regains of genes that had been deleted on the W chromosomes. Together, 14 different genes in the three lineages experienced Z-to-W duplications, with seven of them still functional on the W chromosomes. However, only one of them (*ANXA1*) seems to beneficial to females, therefore its fixation was likely associated with female-specific selection. The other transposed genes might be fixed in the female populations due to the need to restore gene-dosage balance.

## Supplementary Materials

The following are available online at https://www.mdpi.com/2073-4425/11/11/1267/s1, Figure S1: The elevated female SNP density of the ZTR. Figure S2: The coverage and SNP density in both female and male of red bird-of-paradise. Figure S3: Synteny of transposed sequences. Figure S4: Z-to-W transposition of red bird-of-paradise originated at the ancestor of *Paradisaea*. Figure S5: A W-linked 2-kb deletion in red bird-of-paradise transposed region. Figure S6: The expression of *ANXA1* over different developmental stages of the gonads. Figure S7: Two frame shift mutations of *THBS4* on the W-linked transposed sequence. Figure S8: Shared Z-to-W transposition across Darwin’s finches and their close relatives. Figure S9: The Z-to-W transposition in great tit is shared by green backed tit, but not ground tit. Figre S10: Verification of female-specific SNPs in the Z-transposed regions in great tit. Table S1: Accessions of NCBI genome assemblies. Table S2: Resequencing data of the Darwin’s finch and the related species. Table S3: PCR primers used to validate the ZTR in great tit. Table S4: RNA-seq datasets analyzed in this study. Table S5: Divergence rate of the transposed genes measured by pairwise synonymous (dS) and nonnsynonymous (dN) substitution rates between Z/W gametologs.
