# Supplementary material for: Sequence Transpositions Restore Genes on the Highly Degenerated W Chromosomes of Songbirds
Source: Genes (Basel). 2020 Oct 28;11(11):1267. doi: 10.3390/genes11111267 (PMC7692361; doi:10.3390/genes11111267)
Supplement: Supplementary file 1 [file genes-11-01267-s001.zip › supplementary_figures.docx]

**Supplementary Figures**

**
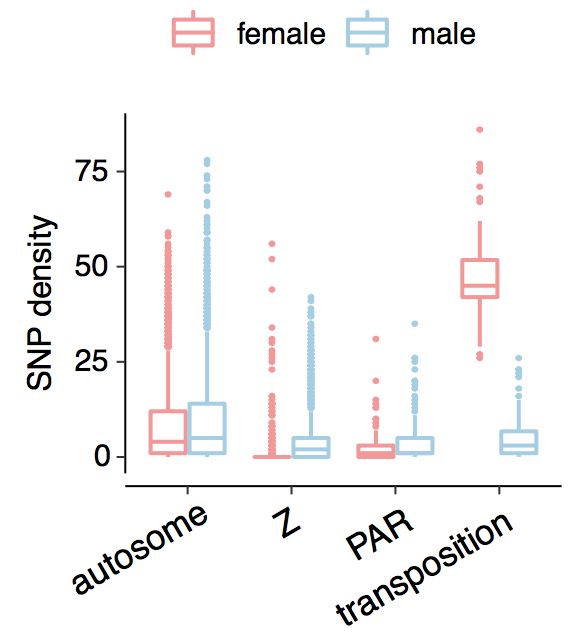
**

**Fig. S1 The elevated female SNP density of the ZTR**. The SNP density was defined as the number of SNPs in 50 kb windows. Most of the Z-linked sequence are hemizygous except for the PAR and ZTR. The ZTR shows a significantly higher SNP density in female than male, due to the divergence of the Z- and W-linked ZTR.

**
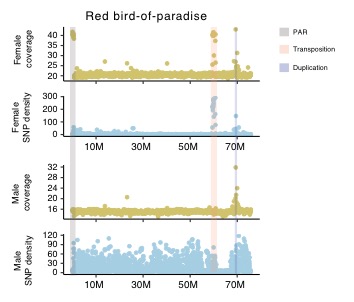
**

**Fig. S2 The coverage and SNP density in both female and male of red bird-of-paradise.** The transposed regions show substantially increased SNP density in female, but not in male, and the coverage level in female become twice of that of other Z-linked sequences. The patterns of coverage and SNP density are different for Z-linked duplications in which male coverage also become twice of the rest Z-linked regions.

**Fig. S3 Synteny of transposed sequences.** Great tit and collared flycatcher have a chromosome assembly. Synteny blocks are shown in grey, while the synteny of individual gene is shown by a red connecting line. We show the synteny of the scaffolds containing transposed sequences to the Z chromosomes of great tit and collared flycatcher. The synteny is analyzed with MCscan (a jcvi tool).

**
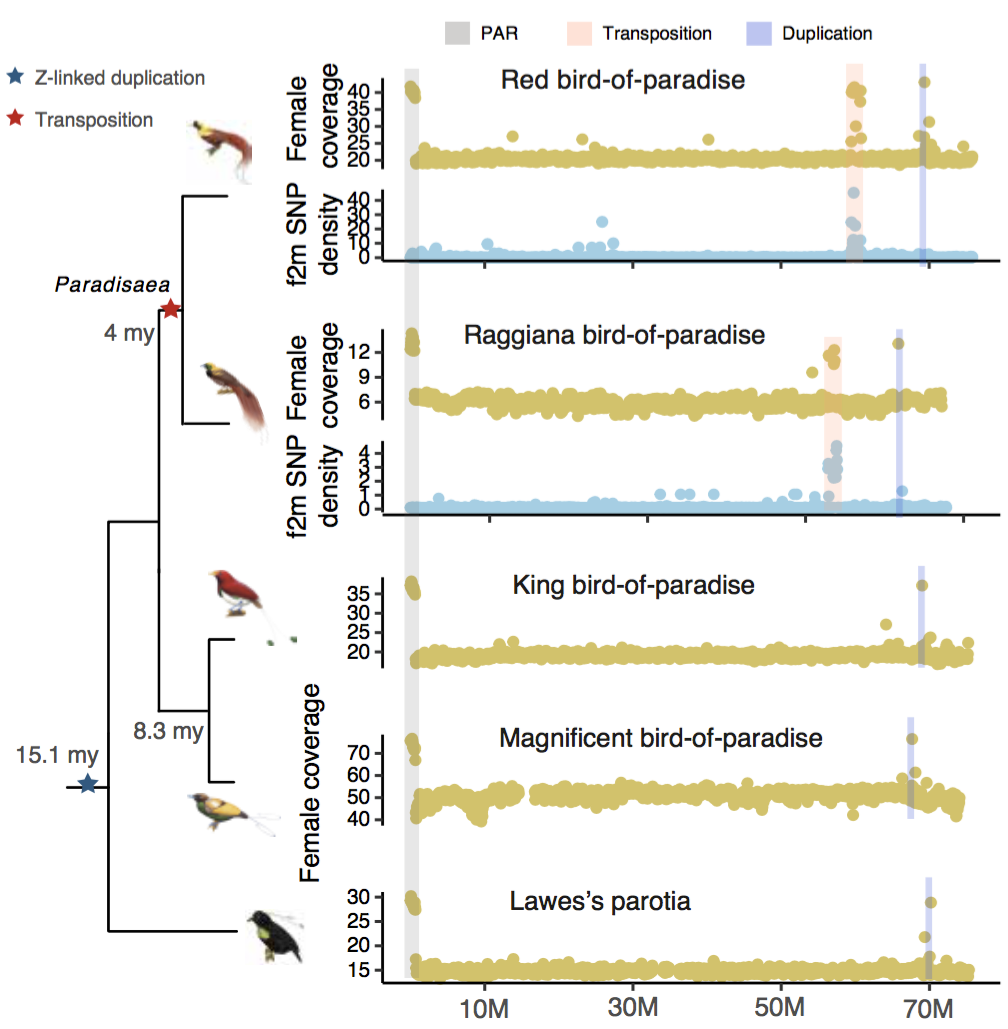
**

**Fig. S4 Z-to-W transposition of red bird-of-paradise originated at the ancestor of *Paradisaea*.** Signals of Z-to-W transpositions were examined in five genomes of birds-of-paradise. The 1.3 Mb transposition discovered in red bird-of-paradise and Raggiana bird-of-paradise are homologous, but are not found in the other species. The origin of the transposition is therefore about 4 my ago when the *Paradisaea* lineage diverged from other birds-of-paradise. The duplication of a ~50k sequence is shared by all species.

**
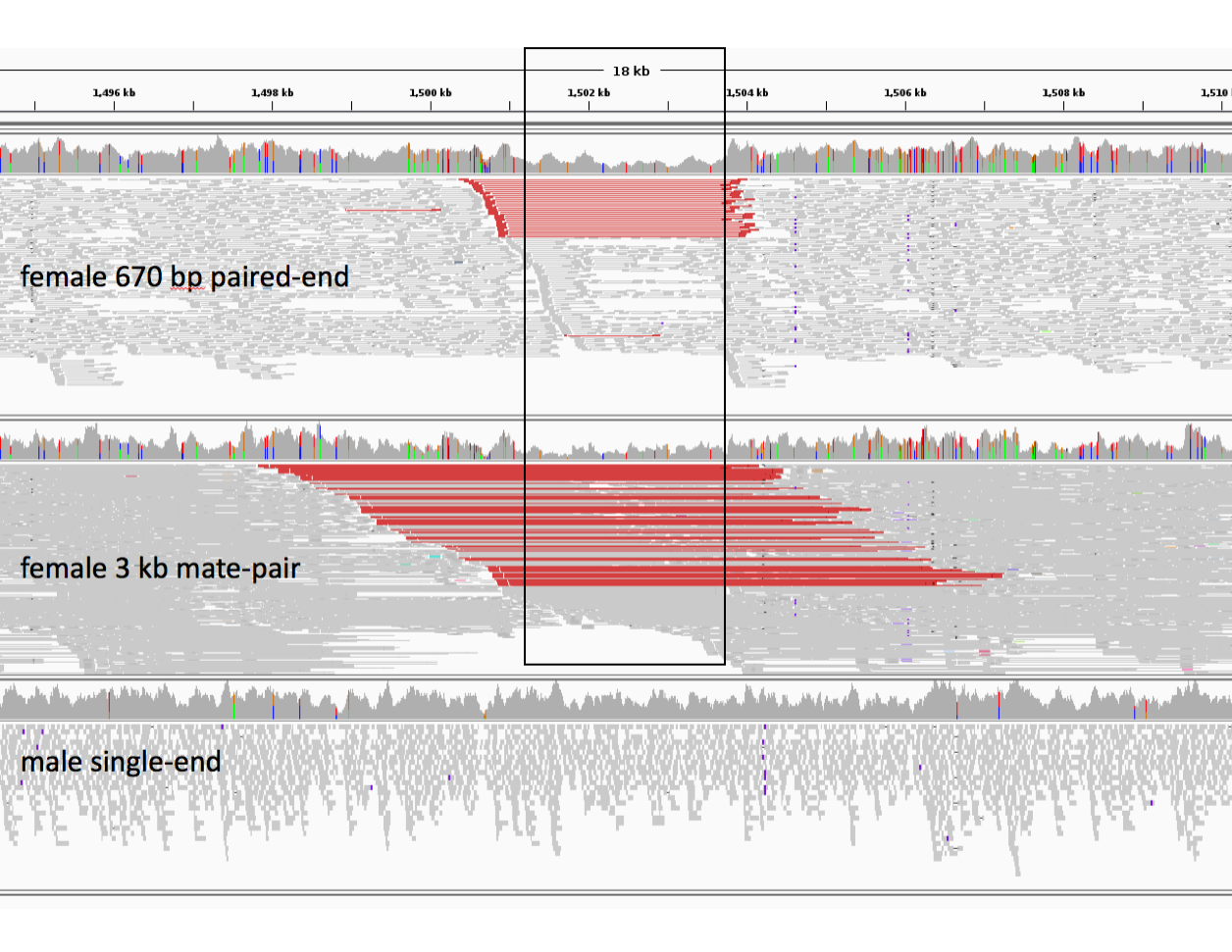
Fig. S5 A W-linked 2-kb deletion in red bird-of-paradise transposed region.** We showed paired-end read pairs with an insert size of 670bp, and mate-pair read pairs with an insert size of 3kb as color-coded lines. Red lines indicated a deletion with a length about 2kb: for example, in the 670bp track, the red read pairs spanned a region over 2kb long. The grey peaks above the read pair tracks showed the heterozygous sites with colored vertical lines, and the heights of peaks indicated the read coverage. At the deleted region, there are reduced levels of heterozygosity and read coverage.

**Fig. S6 The expression of ANAX1 over different developmental stages of the gonads.** The error bars denote the standard deviation of gene expression levels across biological replicates. The number of replicates (n=) were labelled under the labels of the developmental stages on the X-axis. ANAX1 is highly expressed in the adult ovary but not in the testis or gonads of earlier stages.


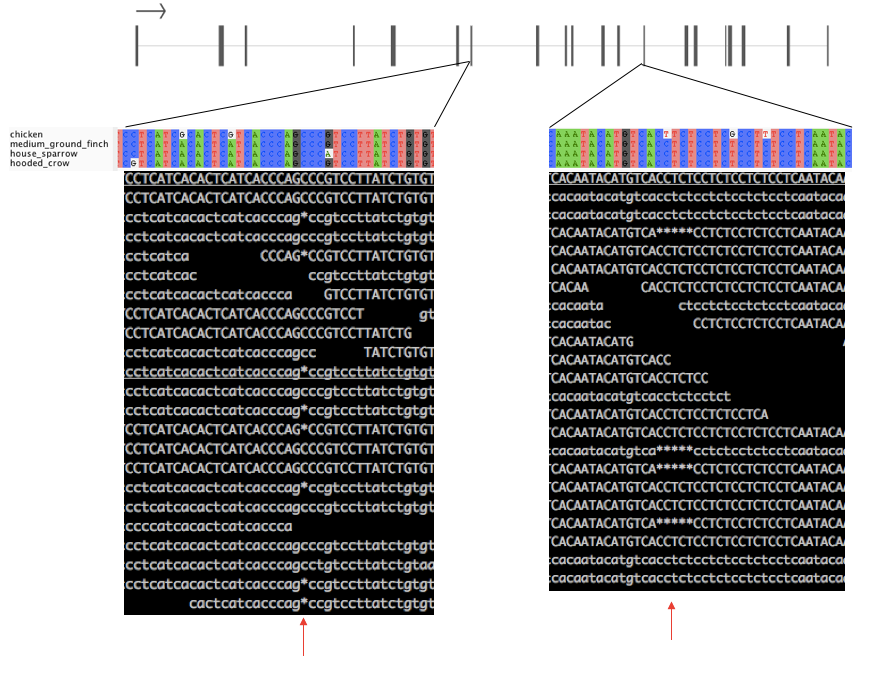


**Fig. S7. Two frame shift mutations of *THBS4* on the W-linked transposed sequence.** The panel in the middle shows the sequence alignments of four birds, and panels below show the alignment of female reads (‘samtools tview’ visualisation). A deletion of one basepair at the 7^th^ exon and a deletion of 5 basepairs at the 13^th^ exon have probably disrupted the open reading frame of the coding sequences.

**
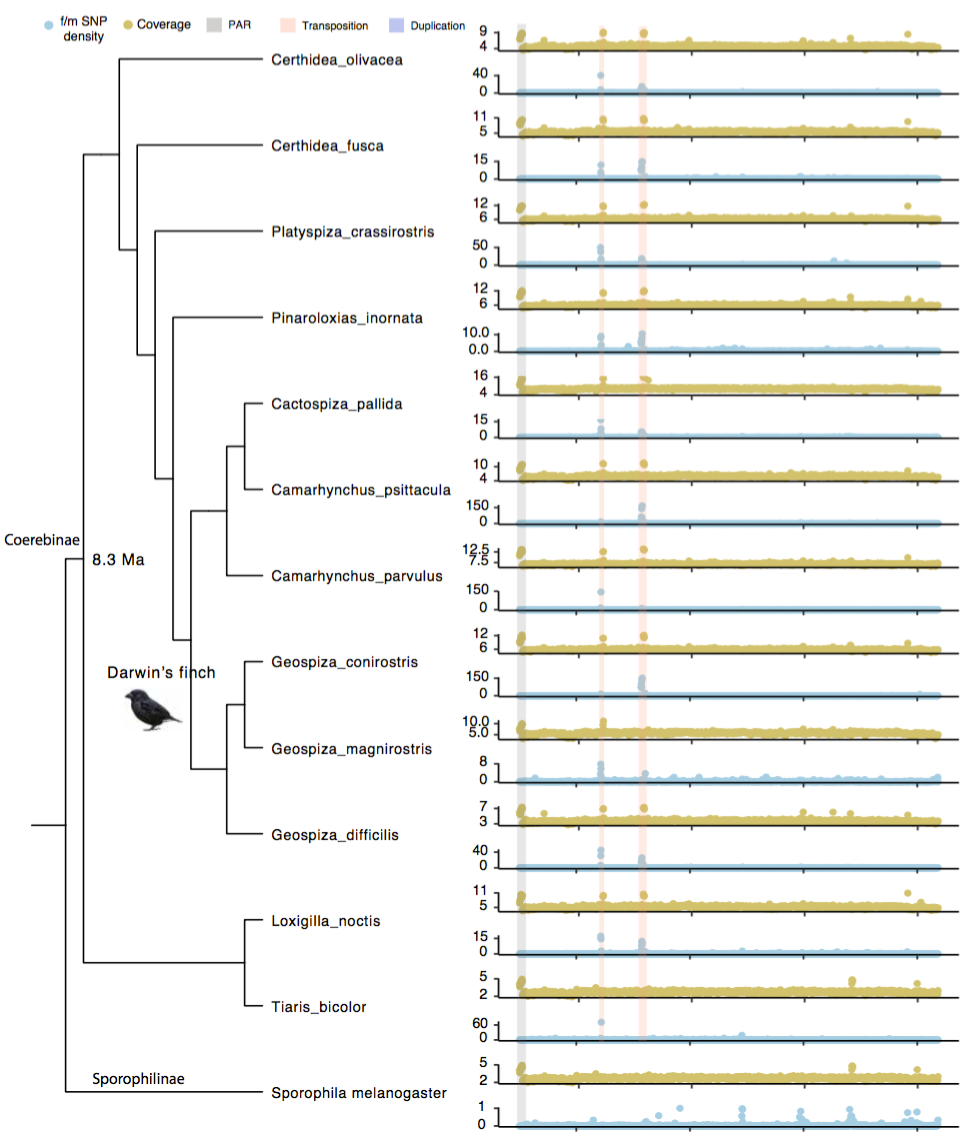
**

**Fig. S8 Shared Z-to-W transposition across Darwin’s finches and their close relatives.** We used the re-sequencing data of 12 Coerebinae (including 6 Darwin’s finches) and one Sporaphilinae species (sister group to Coerebinae) to screen for signals of transpositions. Reads were mapped against the genome of medium ground finch. All Coerebinae species share the two transpositions as seen in medium ground finch, but the *Sporophilia* species does not show the signature of transposition.


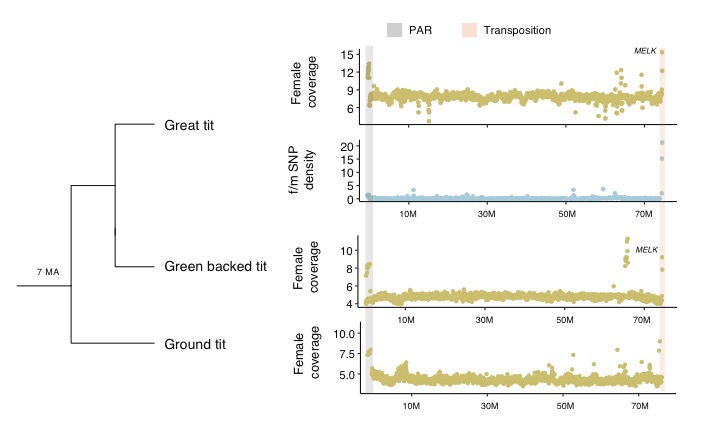


**Fig. S9 The Z-to-W transposition in great tit is shared by green backed tit, but not ground tit**. For green backed tit (*Parus monticolus*), we used the great tit genome as a reference for read mapping. For ground tit, the reference genome GCF_000331425.1 was used, and we built the pseudo-Z chromosome of it based on the Z-chromosome assembly of great tit. Due to a lack of male sequencing data, we were not able to produce f/m SNP density for green backed tit and ground tit, but because the doubled coverage at the *MELK* locus is also seen in green backed tit, it’s likely it shares the same transposition as great tit. The *MELK* locus does not have doubled coverage in ground tit, although a nearby region shows an elevated coverage. The divergence time of great tit and green backed tit is unclear, but because the outgroup species ground tit does not have the *MELK* transposition, we infer the transposition occurred within 7 MA, after divergence of the ancestor of great tit and green backed tit from ground tit.


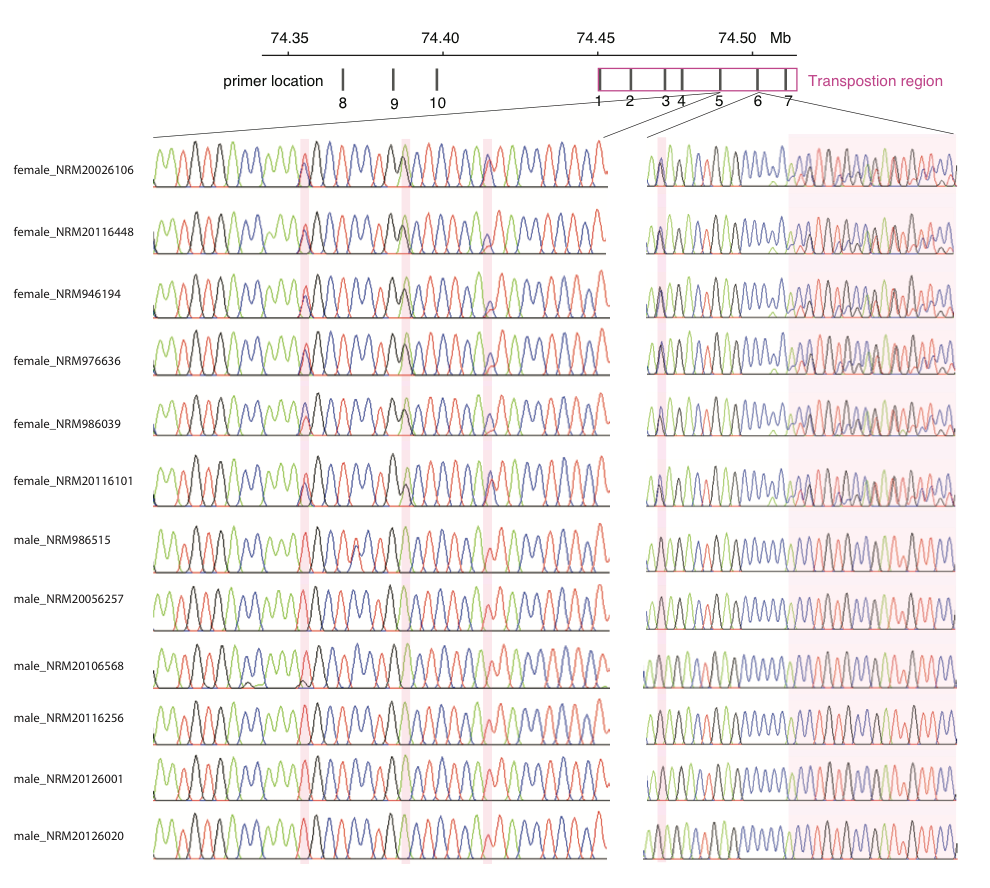


**Fig. S10. Verification of female-specific SNPs in the Z-transposed regions in great tit.** The fragments have been amplified from 6 females and 6 males, with 7 of them at the Z-transposed regions (ZTR) and 3 near the ZTR. One region from the product 5 shows three female-specific SNPs, and one region of the product 6 shows one female-specific SNP and signals of female-specific indels.
